# Utilization of rapid antigen tests for screening SARS-CoV-2 prior to dental treatment

**DOI:** 10.3389/froh.2022.930625

**Published:** 2022-10-04

**Authors:** Pisha Pittayapat, Vorapat Trachoo, Chongpean Jirachoksopon, Kalaya Udom, Chunya Champakerdsap, Oraphan Rungrojwittayakul, Paksinee Kamolratanakul, Pairoj Linsuwanont, Lawan Boonprakong, Natthavoot Koottathape, Vitara Pungpapong, Thanaphum Osathanon, Pornchai Jansisyanont

**Affiliations:** ^1^ Department of Radiology, Faculty of Dentistry, Chulalongkorn University, Bangkok, Thailand; ^2^ Department of Oral and Maxillofacial Surgery, Faculty of Dentistry, Chulalongkorn University, Bangkok, Thailand; ^3^ Faculty of Dentistry, Chulalongkorn University, Bangkok, Thailand; ^4^ Department of Operative Dentistry, Faculty of Dentistry, Chulalongkorn University, Bangkok, Thailand; ^5^ Office of Research Affairs, Faculty of Dentistry, Chulalongkorn University, Bangkok, Thailand; ^6^ Department of Statistics, Chulalongkorn Business School, Chulalongkorn University, Bangkok, Thailand

**Keywords:** SARS-CoV-2, antigen test, dental, COVID-19, patients, triage

## Abstract

Potential aerosols containing severe acute respiratory syndrome coronavirus 2 (SARS-CoV-2) viral particles can be generated during dental treatment. Hence, patient triage is essential to prevent the spread of SARS-CoV-2 in dental clinical settings. The present study described the use of rapid antigen tests for SARS-CoV-2 screening prior to dental treatment in an academic dental clinical setting in Thailand during the pandemic. The opinions of dental personnel toward the use of rapid antigen test screening prior to dental treatment were also assessed. From August 25 to October 3, 2021, dental patients who were expected to receive aerosols generating dental procedures were requested to screen for SARS-CoV-2 using a rapid antigen test before their treatment. A total of 7,618 cases completed the screening process. The average was 212 cases per day. Only five patients (0.07%) were positive for SARS-CoV-2 in the rapid antigen screening tests. All positive cases exhibited mild symptoms. For the questionnaire study, experienced dental personnel frequently and consistently agreed with the use of the rapid antigen test for SARS-CoV-2 screening, which made them feel safer during their patient treatment. However, implementing rapid antigen tests for SARS-CoV-2 may increase the total time spent on a dental appointment. In conclusion, a rapid antigen test could detect the infected individual prior to dental treatment. However, the specificity of rapid antigen tests for SARS-CoV-2 must be taken into account for consideration as a screening process before dental treatment. The enhanced infection control protocols in dental treatment must be consistently implemented.

## Introduction

In December 2019, a report identified a cluster of pneumonia cases in China. These infections were later unveiled upon the intriguing discovery of a new coronavirus, severe acute respiratory syndrome coronavirus 2 (SARS-CoV-2) (1). The respiratory disease caused by the SARS-CoV-2 infection was renamed coronavirus disease 2019 or COVID-19. In Thailand, the first case was reported in January 2020 after a taxi driver exhibited COVID-19 symptoms (2) potentially related to his contact with Chinese tourists while driving them to their destinations. Toward the end of 2020, the emergency use of the SARS-CoV-2 vaccine was approved, aiming to reduce the number and severity of COVID-19 cases. The mRNA vaccine BNT162b2 or mRNA-1273 effectively decreased the infection, particularly the more severe symptoms in individuals with COVID-19 (3). In addition, the vaccination process also prevented the infection according to previous reports, showing that vaccines using ChAdOx1 nCoV (AstraZeneca), BNT162b2 (BioNTech, Pfizer), and mRNA-1273 (Moderna) reduced the percentage of SARS-CoV-2 infection in healthcare workers (4). As of November 10, 2021, the World Health Organization declared four variants of concern, namely, alpha (B.1.1.7), beta (B.1.351), gamma (P.1), and delta (B.1.617.2) (5).

Because SARS-CoV-2 could be detected in the saliva (6), the spread of this coronavirus was identified as a critical concern due to the high potential for transmission in the dental clinic. A study indicated that salivary viral load was higher than nasopharyngeal swab samples in the first week after the onset of symptoms (7). However, the potential infection from contact with saliva loaded with SARS-CoV-2 viral particles requires more evidence to pinpoint its accurate infectivity rates (8). In the dental clinic, both direct and indirect transmission of SARS-CoV-2 can occur. Saliva-contaminated aerosol and droplets generated during dental procedures are potentially considered the source of transmission (9). During the COVID-19 pandemic, oral health care procedures, guidelines, and policies were soon modified in response to the pandemic (10). Dental treatments were limited to emergency cases and urgent care during early pandemic days, and aerosol-generating dental procedures were limited to those deemed strictly necessary (10). Enhanced infection control and prevention protocols were then implemented. In this regard, dental personnel were required to wear enhanced personnel protective equipment, such as N95 respirator masks and waterproof gowns (7, 10). Further, the effective installation and management of air ventilation took place in dental treatment units to reduce the potential for SARS-CoV-2 presence in aerosols generated in these units.

Point-of-care SARS-CoV-2 testing was recommended prior to dental treatment in those cases where the risk is high enough to increase the complexity of dental treatment (11). Point-of-care SARS-CoV-2 testing is one of the potential approaches to control and monitor SARS-CoV-2 infection and transmission during dental care, providing a safe dental treatment environment. A real-time polymerase chain reaction (RT-PCR) is considered the standard diagnostic technique for SARS-CoV-2 detection (11, 12). However, RT-PCR exhibits relevant disadvantages associated with high running costs, and it also requires experienced technicians to run the equipment and long processing times (hours) for each sample.

Another approach for SARS-CoV-2 detection is antigen testing. A rapid antigen test is proposed as a simple screening tool for identifying asymptomatic cases, as the agreement between the rapid antigen test and RT-PCR was high (13). It has been reported in several publications that the rapid antigen test was highly reliable (14). Notably, the cost and processing time are much lower than those for RT-PCR. Hence, the rapid antigen test for SARS-CoV-2 was implemented as the point-of-care test in various clinical settings, including emergency units, for the initial patient screening (15).

The present study described using a rapid antigen test for SARS-CoV-2 prior to dental treatment in a Thai Dental Hospital. Dental personnel were surveyed to evaluate the benefits and limitations of using the rapid antigen screening test prior to dental treatment.

## Materials and methods

This study was approved by the Human Research Ethical Committee of the Faculty of Dentistry at Chulalongkorn University in the province of Bangkok in Thailand (certificate no. 077/2021). The retrospective study was performed in accordance with the ethical standards of the institution. Inform consent was obtained from the participants in the questionnaire study.

### Retrospective study design

From August 25 to October 3, 2021, a retrospective study was performed on dental patients who received the rapid antigen test for SARS-CoV-2 screening prior to their procedures at the dental clinics located in the Faculty's Hospital. Only patients who required aerosol-generating dental procedures were requested to complete rapid antigen screening, which was carried out at the Center for SARS-CoV-2 Rapid Antigen Test, located in the Faculty of Dentistry. Referring patients for SARS-CoV-2 screening also depends on the judgment of dentists. Patients were required to come 1 h before their scheduled dental appointments to take their rapid antigen test, and their screening results were valid for 72 h. A nasopharyngeal swab method was employed to collect samples by trained medical personnel. Three types of rapid antigen test kits were utilized, namely, Biosynex Autotest Antigénique COVID-19 Ag^+^ (Biosynex Swiss SA, Delémont, Jura, Switzerland), Standard Q COVID-19 Ag Test (SD Biosensor Inc., Republic of Korea), and Humasis COVID-19 test (Humasis Co., Ltd., Republic of Korea). All kits were approved by the Thai Food and Drug Administration and available in the market at the time of the fourth wave of the pandemic in Thailand (July–September 2021). At the Center for SARS-CoV-2 Rapid Antigen Test, the biosafety unit installed on a modified truck trailer was donated by the King Mongkut Chaokhun Thahan Hospital Foundation and King Mongkut's Institute of Technology Ladkrabang. The biosafety unit was equipped with a standard controlled air ventilation system featuring a positive air pressure environment with an integrated HEPA filter. Hospital records were used to retrieve each subject's clinical information (age and gender). For those cases that had a positive antigen test, patients were informed to confirm with RT-PCR methods.

### Self-administered questionnaire and outcomes

A self-administered questionnaire was designed by the authors and created online using a Google form format. The consent statement was displayed on the first page of the questionnaire. The first section of the same questionnaire collected sociodemographic data, including working position in the faculty, gender, and age. The second section was composed of five questions (Table 1), and each of them was linked to a response using a 1–5 rating scale (5, strongly agree; 4, agree; 3, neutral; 2, disagree; 1, strongly disagree). The link was distributed to personnel in the dental hospital who were involved both directly and indirectly in dental procedures, including undergraduate (UG) students, dentists, and other personnel (for example, nurses, dental technicians, dental assistants, and hospital staff) at the Faculty of Dentistry Clinics *via* online chat rooms with total members of 1,443. The data were collected from November 11 to December 1, 2021, using the convenience sampling method and approach design. Data were exported into the Excel format and subsequently analyzed.

**Table 1:** Questions displayed in the second section of the self-administered questionnaire.

Statement	Statement description
Statement 1	You agree with the use of the rapid antigen test for SARS-CoV-2 screening of patients before dental treatment
Statement 2	You agree with the use of the rapid antigen test for SARS-CoV-2 screening of dentists and dental personnel before dental treatment
Statement 3	The rapid antigen test for SARS-CoV-2 screening before dental treatment makes you feel safe when performing the dental treatment
Statement 4	The rapid antigen test for SARS-CoV-2 screening before dental treatment does not take much time during dental appointment
Statement 5	The rapid antigen test for SARS-CoV-2 screening before dental treatment does not add complicated steps to dental treatment

### Statistical analysis

A descriptive statistical analysis was first performed. Graphical illustrations were formulated using Prism 9 for MacOS version 9.2.0 (GraphPad Software, San Diego, CA, USA). The descriptive analyses were performed to describe the characteristics of the population in the study. Statistical analysis of questionnaire responses was performed using Wilcoxon's sum rank. A 5% alpha level was set as the statistical cutoff.

## Results

### SARS-CoV-2 screening using the rapid antigen test prior to dental treatment

During the rapid antigen screening period (August 25 to October 3, 2021), the overall number of new daily cases of SARS-CoV-2 and deaths in the province of Bangkok (Thailand), where the faculty is located, gradually decreased. On the first screening day, new confirmed cases were 4,181, and the daily death toll was 99, while on the last screening day, there were 1,221 new cases and 20 deaths.

A total of 7,618 dental patients visiting the Faculty of Dentistry Clinics at the Chulalongkorn University campus required a rapid antigen screening test for SARS-CoV-2 prior to their dental appointments. The average number of dental patients receiving the rapid antigen test was 212 per day. Among these dental patients, 38.7% were males. Further demographic characteristics are described in Table 2. The majority of the cases were aged between 41 and 60 (35.7%) and 61–80 (34.2%) years. The lowest and highest ages of dental patients were 10 and 97 years, respectively.

**Table 2:** Demographic characteristics of cases at the Center for SARS-CoV-2 Rapid Antigen Test.

Demographics	Percentage
Sex	
Male	38.7% (2,945/7,618)
Female	61.3% (4,673/7,618)
Age (range from 10 to 97 years)	
10–20	4.6% (352/7,618)
21–40	23.7% (1,830/7,618)
41–60	35.7% (2,717/7,618)
61–80	34.2% (2,609/7,618)
>80	1.4% (110/7,618)

The Standard Q COVID-19 Ag Test kits were employed in 93.1% (7,093 tests) of dental patients. The Biosynex and Humasis COVID-19 antigen test kits were used in 6.0% (454 cases) and 0.9% (71 cases) cases, respectively. The usage amount of each antigen test kit was linked to the available supply. For Thai nationals, the cost of the rapid antigen test screening for SARS-CoV-2 was paid through the Universal Health Coverage, National Health Security Office (NHSO). Only 56 dental patients (0.73%) paid out of pocket for the screening tests as they did not possess Thai nationality.

Of all 7,618 dental patients, 99.93% tested negative for the COVID-19 rapid antigen test. Five dental patients (0.07%) had a positive result: three females and two males, aged 28–65 years old. Dental patients with positive antigen test results were referred to further confirm such findings with RT-PCR and enrolled into the COVID-19 treatment registry for proper treatment. All positive dental patients were detected with the Standard Q COVID-19 antigen test kits. All positive dental patients exhibited at least one hallmark COVID-19 symptom, such as sore throat, coughing, and stuffy nose.

### Self-administered questionnaire outcomes

A total of 101 responses were obtained. The responses were from undergraduate students (32.7%), dentists/postgraduate students (31.7%), and other faculty personnel (35.6%) (Table 3). Most of the responses were from females (76.2%) and younger adults aged 21–40 years (69.3%).

**Table 3:** Sociodemographic characteristics of self-administered questionnaire responders.

Demographics	No.	Percentage
Total responses	101	100.0
Status		
Undergraduate dental students	33	32.7
Dentists/postgraduate dental students	32	31.7
Other personnel	36	35.6
Sex		
Male	21	20.8
Female	77	76.2
Prefer not to be identified	3	3.0
Age		
21–40	70	69.3
41–60	30	29.7
>60	1	1.0

**Figure 1:**
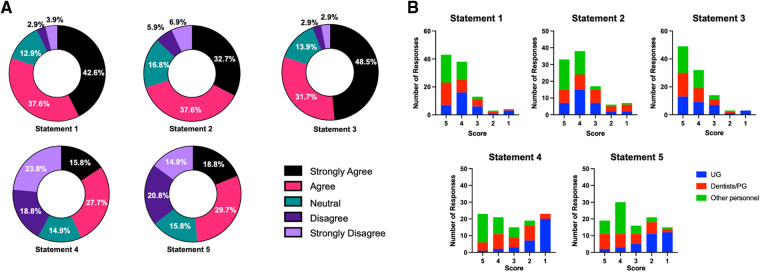
(**A**) percentage of 1–5 rating scale responses for each statement. (**B**) Response numbers for each faculty working status/position (1–5 scale) in each statement.

A summary of responses from self-administered questionnaire outcomes is given in Table 4. For statements 1, 2, and 3, the values of the sum of strongly agree and agree responses were 80.2%, 70.3%, and 80.2%, respectively (Figure 1A), while the values of the sum of strongly agree and agree responses reported for statements 4 and 5 were only 43.5% and 48.5%, respectively. The breakdown analysis of responses according to the working position at the faculty demonstrated that the trend of responses within each position was comparable for statements 1–3 (Figure 1B). However, it was observed that for statements 4 and 5, the majority of undergraduate students stated that they strongly disagree with the less timing and complexity statements associated with the rapid antigen test for SARS-CoV-2 screening. On the other hand, the majority of dentists, postgraduate students, and other dental care personnel indicated an agreement (“strongly agree” and “agree”) with statements 4 and 5 (*p* < 0.05).

**Table 4:** Summary of responses from self-administered questionnaire outcomes.

Statement	Strongly agree (%)	Agree (%)	Neutral (%)	Disagree (%)	Strongly disagree (%)
Statement 1	42.6	37.6	12.9	2.9	3.9
Statement 2	32.7	37.6	16.8	5.9	6.9
Statement 3	48.5	31.7	13.9	2.9	2.9
Statement 4	15.8	27.7	14.9	18.8	23.8
Statement 5	18.8	29.7	15.8	20.8	14.9

## Discussion

The present study reported that only 0.07% of screened subjects using the rapid antigen test prior to their dental treatment had a positive result. After discussion with dental patients who had positive antigen tests, it was found that all of them exhibited at least one hallmark COVID-19 symptom such as sore throat, cough, and stuffy nose; however, all these COVID-19 symptoms were very mild. Phone or online screening for potential COVID-19 signs and symptoms was employed before the dental appointment. The dental personnel in charge of subject screening missed detecting these mild COVID-19 symptoms *via* phone or online interview as all patients must be absent of any symptoms prior to the reservation of dental treatment. A point-of-care screening could benefit COVID-19 symptoms triage prior to dental treatment if a strict and calibrated patient screening protocol is performed by phone and online tools. This could potentially detect all these positive cases before their visits.

In the legal context of Thailand, screening for SARS-CoV-2 is not a standard mandatory requirement for dental patients prior to dental treatment. At the Chulalongkorn University Faculty of Dentistry Dental Clinics, RT-PCR screening for SARS-CoV-2 is required for those patients who need treatment in the operation room. Because the cost of the rapid antigen test is substantially lower than that of the standard RT-PCR, the antigen test was implemented to identify asymptomatic COVID-19 patients. Regardless, the cost of the rapid antigen test was not a relevant financial burden for subjects participating in this study as it was covered by the Universal Health Coverage for all Thai nationals. However, implementing point-of-care screening in other settings, particularly for-profit like private dental practices, may lead to an increase in manpower costs, which may be transferred to the final treatment expense bill of dental patients seen in those settings.

The detection rate of COVID-19-positive dental patients was much lower than the incidence of daily COVID-19 cases seen in Bangkok during the same period. The explanation might be that dental patients are aware of the close contact between the dentist and their oral cavity and the high risk of exposure to potentially contaminated saliva with SARS-CoV-2 viral particles. Therefore, generally speaking, dental patients were cautious about their potential exposure to SARS-CoV-2. Most importantly, in general, those patients who were comfortable seeking dental treatment during the fourth wave of the pandemic in Thailand had received a complete vaccination scheme. However, to make a dental appointment, vaccination was not mandatory for participants/patients enrolled in this study. Thus, the population sample of those who came to our hospital might not be the same as the general population regarding their vaccination status.

It has been reported that sample collection can influence the performance of the rapid antigen test. The rapid antigen test exhibited higher sensitivity for SARS-CoV-2 detection when used with nasopharyngeal swabs than saliva and throat swab samples (16, 17). However, collecting saliva samples could be more convenient and offer more comfort for dental patients, especially pediatric ones. In our school, we employed nasopharyngeal swabs for sample collection to ensure effective SARS-CoV-2 detection.

The rapid antigen test can be used in population-based screening to identify asymptomatic individuals and control the spread of SARS-CoV-2 viral infection (18). However, the rapid antigen test provides less sensitivity and specificity than the RT-PCR technique. The lower sensitivity of rapid antigen kit tests is of paramount concern, and the sensitivity varies widely among different kit types and company brands (19). Despite these challenges, the specificity is higher in those patients with a higher viral load (19). False-negative outcomes were reported when rapid antigen tests were employed at a pediatric emergency unit in Italy, although those were at a very low incidence (15). In addition, the current rapid antigen kit may not be able to detect more recent SARS-CoV-2 variants like Omicron, among others arising. These challenges can be seen as a major disadvantage of rapid antigen tests as a SARS-CoV-2 screening tool prior to dental treatment.

Recently, it has been reported that rapid antigen tests can be helpful for the initial screening of patients in emergency units (15). However, it should be noted that in asymptomatic patients, the outcomes of rapid antigen tests cannot be utilized to rule out a positive infection status because false-negative results are often common (20). In the present study, the limited supply of antigen test kits during the fourth wave of the COVID-19 pandemic in Thailand led our team to take an efficacious strategy and practical approach to using such limited kits in this study. Those patients who are set up to receive aerosol-generating dental treatment have a higher chance of spreading viral particles in the dental treatment environment (21, 22). Thus, the screening for SARS-CoV-2 infection was focused on these groups, despite the fact that all patients possess the risk of infection. Therefore, the point-of-care use of rapid antigen for SARS-CoV-2 detection in dental settings cannot exclude other preventive protocols prior to dental treatment. For example, a calibrated, reliable, and validated phone and online screening process, proper personnel protective equipment, the use of pre-operative mouthwash, the establishment of air-flow modifications, surface disinfection, and common universal infection control protocols should be utilized and implemented as a unified standard operating procedure.

During the data collection period, the dental personnel were not required for rapid antigen test screening prior to duty due to the limited supply available. In the dental treatment procedure, all dental personnel were strictly required to wear personal protective equipment, including N95 masks. Hence, the risk for transmission was relatively low. However, dental personnel could request rapid antigen test screening if they were suspected of COVID19-related symptoms or in close contact with the SARS-CoV-2 positive cases.

The overall perception of faculty personnel (UG students, graduate students, dentists, and other auxiliary staff) regarding the use of the rapid antigen test for SARS-CoV-2 screening exhibited a striking agreement upon its use on both patients and dental personnel prior to dental treatment sessions. The faculty personnel considered that this screening test could make them feel safer during dental appointments. The majority of undergraduate students strongly disagree with the statement that the rapid antigen test for SARS-CoV-2 screening does not time-consuming and is less complex. However, the rapid antigen test screening was performed by trained medical personnel, and patients were asked to come 1 h prior to their dental appointment. The registration steps for hospital records and the reimbursement from the Universal Health Coverage caused an accumulation of patients at the Center for SARS-Cov-2 Rapid Antigen Test, leading to the delay in the early morning (8.00 am–10.00 am) and afternoon (12.30 pm–2.00 pm) sessions. Most undergraduate students are concerned that such screening adds additional steps and more time constraints during dental appointments since these students are heavily involved and committed to patient dental care to be able to graduate successfully.

## Limitations and conclusion

The present study did not collect the vaccination status and/or antibody levels against SARS-CoV-2 of those patients who had undergone antigen test screening. This information could help to understand the observation of a low percentage of SARS-CoV-2 antigen test positive prior to dental treatment during the period of screening in this project. In addition, the current observation employed three types of antigen test kits. Although all the kits passed the standard and were certified by the Thai Food and Drug Administration, they might exhibit a slight difference in specificity and sensitivity, and this point should also be considered when interpreting the results. In addition, for the self-administered questionnaire, the survey was sent to 1,433 people, but only 101 responses were obtained; hence, the interpretation of the outcomes may be performed with the caution noted that this sampling may not directly represent the targeted population.

The present study reported low incidence rates of SARS-CoV-2 with three rapid antigen screening tests prior to dental treatment. All positive dental patients had mild symptoms related to COVID-19. Hence, a standard calibrated phone/online patient screening procedure prior to dental appointments is deemed essential to exclude potentially infected individuals. The implementation of rapid antigen tests for SARS-CoV-2 as a point-of-care screening method should be carefully considered due to the additional costs for the national health care system and clinical settings. With vaccination coverage and decreasing severe symptom cases, the consideration for general screening prior to dental procedures might not be necessary. In addition, the low specificity of rapid antigen tests for SARS-CoV-2 must be considered for appropriate decision-making when aiming to achieve an effective screening process (15, 19, 20). On top of patient triage protocols, dentists should be aware of and updated on the aseptic techniques to prevent the transmission of COVID-19 in dental clinics (23). Enhanced infection control procedures and proper aerosol management in each dental unit should be consistently performed as an overall standardized operational procedure for each clinical setting.

## Data Availability

The raw data supporting the conclusions of this article will be made available by the authors, without undue reservation.

## References

[1] Huang C Wang Y Li X Ren L Zhao J Hu Y Clinical features of patients infected with 2019 novel coronavirus in Wuhan, China. Lancet. (2020) 395(10223):497–506. 10.1016/S0140-6736(20)30183-5 31986264 PMC7159299

[2] Pongpirul WA Pongpirul K Ratnarathon AC Prasithsirikul W. Journey of a Thai taxi driver and novel coronavirus. N Engl J Med. (2020) 382(11):1067–8. 10.1056/NEJMc2001621 32050060 PMC7121480

[3] Chung H He S Nasreen S Sundaram ME Buchan SA Wilson SE Effectiveness of BNT162b2 and mRNA-1273 COVID-19 vaccines against symptomatic SARS-CoV-2 infection and severe COVID-19 outcomes in ontario, Canada: test negative design study. Br Med J. (2021) 374:n1943. 10.1136/bmj.n1943 34417165 PMC8377789

[4] Paris C Perrin S Hamonic S Bourget B Roue C Brassard O Effectiveness of mRNA-BNT162b2, mRNA-1273, and ChAdOx1 nCoV-19 vaccines against COVID-19 in healthcare workers: an observational study using surveillance data. Clin Microbiol Infect. (2021) 27:1699.e5–1699.e8. 10.1016/j.cmi.2021.06.043 PMC8275842 34265462

[5] World Health Organization. Tracking SARS-CoV-2 variants. https://wwwwhoint/en/activities/tracking-SARS-CoV-2-variants/. accessed on November 10, 2021.

[6] Bruxvoort K Tenggardjaja CF Slezak J Gullett JC Broder B Park CH Variation in SARS-CoV-2 molecular test sensitivity by specimen types in a large sample of emergency department patients. Am J Emerg Med. (2021) 50:381–7. 10.1016/j.ajem.2021.08.034 34478943 PMC8367656

[7] Chowdhry A Kapoor P Kharbanda OP Popli DB. Saliva and COVID 19: current dental perspective. J Oral Maxillofac Pathol. (2021) 25(1):18–21. 10.4103/jomfp.jomfp_63_21 34349404 PMC8272509

[8] Jansen GJ Wiersma M van Wamel WJB Wijnberg ID. Direct detection of SARS-CoV-2 antisense and sense genomic RNA in human saliva by semi-autonomous fluorescence in situ hybridization: a proxy for contagiousness? PLoS One. (2021) 16(8):e0256378. 10.1371/journal.pone.0256378 34403446 PMC8370601

[9] Inchingolo AD Inchingolo AM Bordea IR Malcangi G Xhajanka E Scarano A SARS-CoV-2 disease through viral genomic and receptor implications: an overview of diagnostic and immunology breakthroughs. Microorg. (2021) 9(4):793. 10.3390/microorganisms9040793 PMC8070527 33920179

[10] Jiang CM Duangthip D Auychai P Chiba M Folayan MO Hamama HH Changes in oral health policies and guidelines during the COVID-19 pandemic. Front Oral Health. (2021) 2:668444. 10.3389/froh.2021.668444 35048011 PMC8757803

[11] Shirazi S Stanford CM Cooper LF. Testing for COVID-19 in dental offices: mechanism of action, application, and interpretation of laboratory and point-of-care screening tests. J Am Dent Assoc. (2021) 152(7):514–25. 10.1016/j.adaj.2021.04.019 34176567 PMC8096195

[12] Lee J Song JU Shim SR. Comparing the diagnostic accuracy of rapid antigen detection tests to real time polymerase chain reaction in the diagnosis of SARS-CoV-2 infection: a systematic review and meta-analysis. J Clin Virol. (2021) 144:104985. 10.1016/j.jcv.2021.104985 34560340 PMC8444381

[13] Kyritsi M Vontas A Voulgaridi I Matziri A Komnos A Babalis D Rapid test ag 2019-nCoV (PROGNOSIS, BIOTECH, larissa, Greece); performance evaluation in hospital setting with real time RT-PCR. Int J Environ Res Public Health. (2021) 18(17):9151. 10.3390/ijerph18179151 34501741 PMC8431120

[14] Stokes W Berenger BM Singh T Adeghe I Schneider A Portnoy D Acceptable performance of the Abbott ID NOW among symptomatic individuals with confirmed COVID-19. J Med Microbiol. (2021) 70(7):001372. 10.1099/jmm.0.001372 PMC8493423 34309503

[15] Denina M Giannone V Curtoni A Zanotto E Garazzino S Urbino AF Can we trust in Sars-CoV-2 rapid antigen testing? Preliminary results from a paediatric cohort in the emergency department. Ir J Med Sci. (2021) 191:1767–70. 10.1007/s11845-021-02776-z 34519927 PMC8438652

[16] Kritikos A Caruana G Brouillet R Miroz JP Abed-Maillard S Stieger G Sensitivity of rapid antigen testing and RT-PCR performed on nasopharyngeal swabs versus saliva samples in COVID-19 hospitalized patients: results of a prospective comparative trial (RESTART). Microorganisms. (2021) 9(9):1910. 10.3390/microorganisms9091910 34576805 PMC8464722

[17] Stokes W Berenger BM Portnoy D Scott B Szelewicki J Singh T Clinical performance of the Abbott panbio with nasopharyngeal, throat, and saliva swabs among symptomatic individuals with COVID-19. Eur J Clin Microbiol Infect Dis. (2021) 40(8):1721–6. 10.1007/s10096-021-04202-9 33742322 PMC7979467

[18] Martin-Sanchez V Fernandez-Villa T Carvajal Uruena A Rivero Rodriguez A Reguero Celada S Sanchez Antolin G Role of rapid antigen testing in population-based SARS-CoV-2 screening. J Clin Med. (2021) 10(17):3854. 10.3390/jcm10173854 34501297 PMC8432187

[19] Andreani J Lupo J Germi R Laugier C Roccon M Larrat S Evaluation of six commercial SARS-CoV-2 rapid antigen tests in nasopharyngeal swabs: better knowledge for better patient management? J Clin Virol. (2021) 143:104947. 10.1016/j.jcv.2021.104947 34492569 PMC8376530

[20] Shaw JLV Deslandes V Smith J Desjardins M. Evaluation of the Abbott panbio(TM) COVID-19 ag rapid antigen test for the detection of SARS-CoV-2 in asymptomatic Canadians. Diagn Microbiol Infect Dis. (2021) 101(4):115514. 10.1016/j.diagmicrobio.2021.115514 34418823 PMC8324400

[21] Baldion PA Rodriguez HO Guerrero CA Cruz AC Betancourt DE. Infection risk prediction model for COVID-19 based on an analysis of the settlement of particles generated during dental procedures in dental clinics. Int J Dent. (2021) 2021:7832672. 10.1155/2021/7832672 34976064 PMC8717047

[22] Ou Q Placucci RG Danielson J Anderson G Olin P Jardine P Characterization and mitigation of aerosols and spatters from ultrasonic scalers. J Am Dent Assoc. (2021) 152(12):981–90. 10.1016/j.adaj.2021.06.007 34538418 PMC9671395

[23] Marya A Karobari MI Selvaraj S Adil AH Assiry AA Rabaan AA Risk perception of SARS-CoV-2 infection and implementation of Various protective measures by dentists across Various countries. Int J Environ Res Public Health. (2021) 18(11):5848. 10.3390/ijerph18115848 34072456 PMC8199051

